# Somatostatin Analogues in the Treatment of Neuroendocrine Tumors: Past, Present and Future

**DOI:** 10.3390/ijms20123049

**Published:** 2019-06-22

**Authors:** Anna Kathrin Stueven, Antonin Kayser, Christoph Wetz, Holger Amthauer, Alexander Wree, Frank Tacke, Bertram Wiedenmann, Christoph Roderburg, Henning Jann

**Affiliations:** 1 Charité, Campus Virchow Klinikum and Charité, Campus Mitte, Department of Hepatology and Gastroenterology, Universitätsmedizin Berlin, 10117 Berlin, Germany; anna-kathrin.stueven@charite.de (A.K.S.); antonin.kayser@charite.de (A.K.); alexander.wree@charite.de (A.W.); frank.tacke@charite.de (F.T.); bertram.wiedenmann@charite.de (B.W.); henning.jann@charite.de (H.J.); 2 Charité, Campus Virchow Klinikum and Charité, Campus Mitte, Department of Nuclear Medicine, Universitätsmedizin Berlin, 10117 Berlin, Germany; christoph.wetz@charite.de (C.W.); holger.amthauer@charite.de (H.A.)

**Keywords:** neuroendocrine tumor, carcinoid, somatostatin analogue, octreotide, lanreotide, PROMID, CLARINET, PRRT, NETTER-1

## Abstract

In recent decades, the incidence of neuroendocrine tumors (NETs) has steadily increased. Due to the slow-growing nature of these tumors and the lack of early symptoms, most cases are diagnosed at advanced stages, when curative treatment options are no longer available. Prognosis and survival of patients with NETs are determined by the location of the primary lesion, biochemical functional status, differentiation, initial staging, and response to treatment. Somatostatin analogue (SSA) therapy has been a mainstay of antisecretory therapy in functioning neuroendocrine tumors, which cause various clinical symptoms depending on hormonal hypersecretion. Beyond symptomatic management, recent research demonstrates that SSAs exert antiproliferative effects and inhibit tumor growth via the somatostatin receptor 2 (SSTR2). Both the PROMID (placebo-controlled, prospective, randomized study in patients with metastatic neuroendocrine midgut tumors) and the CLARINET (controlled study of lanreotide antiproliferative response in neuroendocrine tumors) trial showed a statistically significant prolongation of time to progression/progression-free survival (TTP/PFS) upon SSA treatment, compared to placebo. Moreover, the combination of SSA with peptide receptor radionuclide therapy (PRRT) in small intestinal NETs has proven efficacy in the phase 3 neuroendocrine tumours therapy (NETTER 1) trial. PRRT is currently being tested for enteropancreatic NETs versus everolimus in the COMPETE trial, and the potential of SSTR-antagonists in PRRT is now being evaluated in early phase I/II clinical trials. This review provides a synopsis on the pharmacological development of SSAs and their use as antisecretory drugs. Moreover, this review highlights the clinical evidence of SSAs in monotherapy, and in combination with other treatment modalities, as applied to the antiproliferative management of neuroendocrine tumors with special attention to recent high-quality phase III trials.

## 1. Introduction and Basic Pharmacology

The cyclic peptide hormone somatostatin (SST) occurs naturally in the body of mammals, primarily in the central nervous system, the pancreas, and the gastrointestinal (GI) tract [1,2,3,4]. It possesses a wide range of inhibitory functions, including the inhibition of hypothalamic hormones [5,6], the regulation of gastrin and gastric acid secretion [7], the release of insulin, glucagon [8,9], pancreatic amylase [10], and other hormones in the GI tract like cholecystokinin, vasoactive intestinal peptide, and secretin [1]. Furthermore, it can exert inhibitory effects on cell proliferation [11,12,13,14]. Even anti-inflammatory and anti-nociceptory effects have been described [15]. These properties make somatostatin a potential candidate for therapeutic use in a vast range of diseases. Currently, it is used to treat acromegaly, Cushing’s disease, certain forms of upper gastrointestinal bleeding, and neuroendocrine tumors (NETs) [15].

Somatostatin was first discovered in 1973 by Brazeau et al. in an ovine hypothalamic extract at the Salk Institute in La Jolla [16,17,18]. At first, only the 14 aa isoform had been found; the existence of a second, larger isoform was discovered in 1980 [19,20]. Somatostatin is encoded by a gene that lies on chromosome 3q28 [21]. Its production entails two steps: A large precursor peptide called preprosomatostatin is cleaved and converted into prosomatostatin, from which, in turn, the two abovementioned active isoforms of somatostatin are produced [22,23]. Their functions strongly overlap, but the shorter one occurs much more frequently while the larger one shows a higher potency [22]. They have a very short half-life of around 3 min, which initially limited their pharmacological use in the treatment of diseases; longer-lasting somatostatin analogues had to be developed; the first one was octreotide [24]. Over the years, many more were added to the therapeutic arsenal, including lanreotide, vapreotide, seglitide, and pasireotide, with half-lives of 1.5 to 2 h [25]. 

The effect somatostatin has on different cells in different tissues is in part determined by the types of somatostatin receptors expressed on their surface. In 1992, the first two somatostatin receptors were discovered, termed SSTR1 and SSTR2 [26]. Today, 5 SST receptors are known to exist, termed SSTR1 through 5, which are encoded by five different genes, each on a separate chromosome. All are G-protein-coupled receptors [27]. Both isotypes of somatostatin, as well as another endogenous peptide called cortistatin [28], bind to these receptors with similar high affinity, one exception being the even higher affinity of SSTR5 to SST-28 [29,30]. Of these receptors, the SSTR2 is the only one of which there are 2 isoforms, SSTR2a and SSTR2b, produced by alternate splicing [1]. The SSTRs are widely distributed throughout all tissues in the human body, with varying expression profiles of the different receptors. A multitude of intracellular pathways following activation of the receptors have been described. The antisecretory effects are mainly achieved by an inhibition of exocytosis, for example, through altering the levels of second messengers, like cAMP, or by activating ion channels, and thus altering intracellular calcium levels. The antiproliferative effects are achieved by inducing cell cycle arrest or apoptosis through protein tyrosine phosphatases or possibly by inhibiting the release of growth factors [2,15]. The issue is further complicated by the fact that the receptors can form dimers, thereby gaining unique pharmacological properties [22]. There are a number of detailed reviews on somatostatin receptor signaling [1,6,31,32].

There are characteristic receptor profiles of certain tumors—neuroendocrine tumors have repeatedly been shown to express the somatostatin receptors 2 and 5 [15,33,34,35,36], which makes them targets for therapy via certain somatostatin analogues. The intricacies of anti-secretory and antiproliferative usage of SSAs will be discussed in further detail in the following chapters.

## 2. Antisecretory Therapy

In many patients with NETs, hypersecretion of neuropeptides represents a major clinical problem [37]. Carcinoid syndrome (CS) was described for the first time in a case report and has later been associated with the production of serotonin (5-hydroxytryptamine) and histamine by the tumor [38,39,40]. CS occurs in 20% of all NET patients, with the majority of patients displaying a midgut primary [37]. The most common clinical symptoms, caused by tumor-related hormone hypersecretion, are diarrhea and cutaneous flush. Cardiac fibrosis, leading to a carcinoid heart disease, represents a severe and potentially life-threatening complication. While diarrhea and fibrosis are caused by serotonin, flushing and respiratory symptoms are caused by co-secreted peptides such as histamine, bradykinins, and tachykinins [41]. Though curative surgery in neuroendocrine tumors is possible, the CS is mostly associated with liver metastases and, therefore, the treatment-options are non-curative in most cases. Palliative therapeutic options are, following current guidelines, somatostatin analogues (SSAs), interferon alpha, chemotherapy, locoregional therapies, molecular-targeted therapies, and peptide receptor radionuclide therapy (PRRT) [42]. Though, in many cases, these therapies do not stop tumor growth, they are able to improve health related quality of life by reducing the symptoms of CS in these patients [43]. Of note, a recent meta-analysis demonstrated that octreotide reduced diarrhea and flush in 65% and 72% of patients, respectively. Notably, lanreotide showed very similar effects [44]. In patient’s refractory to a standard SSA first-line treatment, dose escalation, or decreasing the injection interval to 21 days, resulted in a reduction of diarrhea in 72% and of flush in 84% of patients [45,46,47,48,49]. 

As mentioned above, patients with CS are also at risk for carcinoid heart disease (CHD) [50]. CHD is characterized by fibrosis of the endocardium and the valve leaflets of the right heart. Since CHD mainly occurs in patients with high levels of serotonin and 5-HIAA [51], treatment with SSA was suggested as a treatment option. In line with this hypothesis, SSA treatment was demonstrated to reduce rates of CHD in patients suffering from CS in a recent trial [52].

In the evolution of add-on treatment-strategies, interferon alpha, first mentioned in 1983, plays an important role due to its antiproliferative and anti-secretory effects. Both in combination with SSA and used as single substance, interferon alpha was demonstrated to provide hormonal control and to reduce clinical symptoms of CS [53,54]. This positive effect is restricted by adverse side effects, such as flu-like symptoms or fatigue and, therefore, interferon alpha nowadays is used less frequently.

Besides interferon alpha, pasireotide was recently tested as a second-line therapy in CS patients refractory to SSA. In these patients, pasireotide led to symptom control in 27% of patients [55]. However, so far, pasireotide has not proven superior to octreotide or lanreotide in antisecretory or antiproliferative therapy. The toxicity profile of pasireotide is also less favorable, since, in addition to gastrointestinal symptoms (mainly abdominal pain, nausea, and diarrhea), which are comparable to first-generation SSAs, a higher incidence of hyperglycemia has been observed [56]. Pasireotide, therefore, currently does not represent a standard of care in patients with NETs. Its hyperglycemic effects might be relevant in selected cases of insulinoma [57] and in NETs with specific SSTR profiles. Recently, a role for telotristat ethyl, an inhibitor of tryptophan hydroxylase, in the treatment of CS was suggested by many authors [58,59]. Telotristat ethyl was shown to significantly reduce diarrhea when used as add-on treatment to SSAs in patients with refractory diarrhea [60]. It has been associated with various side effects including nausea, headache, elevated liver enzymes, depression, peripheral edema, flatulence, decreased appetite, and fever. All of these are reversible after end of treatment and manageable in most patients.

## 3. Antiproliferative Therapy

Up to 90% of gastroenteropancreatic (GEP) NETs carry somatostatin receptors on the membrane and are therefore considered candidates for SSA-based therapy [61]. While initially used in the treatment of carcinoid syndrome to inhibit the release of neuropeptides or bioactive amines, several trials meanwhile revealed an effect of SSAs on tumor cell proliferation. Since their introduction, multiple phase II trials, as well as case series, demonstrated high rates of disease stabilization upon treatment with SSAs and suggested that SSA treatment may prolong both overall and progression-free survival in NETs. In line, data from the US-based Surveillance, Epidemiology, and End Results (SEER) database suggested significantly better outcomes of NET patients treated between 1988 and 2004, when compared to those treated between 1973 and 1987, most probably associated with the introduction of octreotide into the treatment of patients with NETs [62]. Although, in all of these trials, objective radiological responses, potentially leading to a prolonged survival, were only rarely reported (<5% of cases), almost 50% of all patients displayed tumor stabilization [63]. Arnold et al. reported the outcome of 103 patients with metastatic NET who were treated with octreotide (600 μg daily) until objective disease progression or unacceptable toxicity [64]. Despite this, the study failed to detect objective tumor responses in the whole cohort within the subgroup of patients showing stable disease when treatment was started; disease stability lasting >12 months was observed in almost 55% of patients [65]. In further studies, the antitumor activity of intramuscular lanreotide was tested in 46 patients with pancreatic NETs. Out of these, 4% displayed an objective response, while 41% displayed disease stabilization [66] and similar results (7% partial response, 81% stable disease) were reported from another phase II study including GEP-NETs [67]. Considering the limitations of these early trials, the PROMID (placebo-controlled, prospective, randomized study in patients with metastatic neuroendocrine midgut tumors) and the CLARINET (controlled study of lanreotide antiproliferative response in neuroendocrine tumors) studies were conducted in patients with midgut and gastroenteropancreatic NETs, respectively [68,69]. Within the PROMID trial, 85 patients with well-differentiated NETs received either octreotide or placebo. The primary endpoint was time to tumor progression. Octreotide was associated with a significant longer time to tumor progression compared to the placebo (14.3 month within the octreotide group and 6.0 months in the placebo group). At 6 months, significantly lower tumor progression rates were observed within the octreotide arm (24 %vs 66%), highlighting the antiproliferative effect of SSAs. Patients with lower tumor burden and resected primary tumor displayed a more favorable outcome. CLARINET tested the somatostatin analogue lanreotide in patients with advanced, G1/G2 differentiated, nonfunctioning, somatostatin receptor–positive NETs and documented disease progression status. The primary endpoint was progression-free survival (PFS). The administration of lanreotide was linked to significantly prolonged PFS compared to the placebo (estimated rates of progression-free survival at 24 months 65.1% in the lanreotide group and 33.0% in the placebo group). Long-term results from both trials have been reported only recently (Table 1 and Table 2) [70,71].

Meanwhile, different authors suggested that an escalation of SSA dosage might further improve antiproliferative/antitumor effects. However, only a small number of studies showing (at least partially) contradictory results are available. In a recent meta-analysis, 18 trials using more than 30 mg octreotide or 120 mg lanreotide over 28 days (1022 patients), have been included [72]. Using higher SSA doses, response rates remained modest (0–14%) and, despite the encouraging disease control rates that could be achieved, large, prospective studies are needed to finally answer the question regarding the role of escalated-dose SSA treatment in patients with NET. 

Conventional SSAs specifically bind the somatostatin receptor 2. By contrast, pasireotide, a novel multireceptor-targeted somatostatin displays a broader spectrum and additionally binds SSTR1, 3, and 5 [73]. Preclinical data from NCI-H727 cells, as well as from pancreatic NET primary cell cultures, revealed a more potent antiproliferative effect of pasireotide compared to octreotide [74]. Notably, these data could be reproduced in mice, thus providing robust evidence for a clinical use of pasireotide [75,76]. These preclinical data gave rise to the hope that pasireotide represents a more effective antiproliferative tool in the treatment of patients with NETs but results from clinical trials have, so far, not been as positive as expected. Cives et al. recently demonstrated that pasireotide LAR provides long-lasting tumor control efficacy (PFS 11 months), when used as first-line therapy in patients with advanced NET [77]. Moreover, in patients with functionally active advanced GEP-NETs, refractory to available SSA treatment, pasireotide provided an improved tumor control rate at 6 months compared to octreotide [56]. Combinations of pasireotide with different substances (e.g., cabergoline, aurora, teriflunomide) have recently be analyzed. Most prominently, the COOPERATE-2 trial tested the combination of everolimus and pasireotide vs. everolimus in 160 patients with progressive grade 1 through 2 pancreatic NETs [78]. Unexpectedly, both overall and progression-free survival were similar in both arms (16.8 months vs. 16.6 months), although response rates were higher in the experimental arm. Notably, similar results were shown by the phase II LUNA trial, highlighting that the use of such combinations should be limited to clinical trials [79]. 

## 4. Peptide Receptor Radionuclide Therapy (PRRT)

The high-level expression of somatostatin receptors (SSRs) 2 and 5 on the tumor cell surface in the majority of neuroendocrine tumors (NETs) provides the basis not only for sensitive functional imaging, but also for a tumor-targeted therapy with commonly “cold”, as well as radioisotope-labeled “hot” somatostatin analogues [80]. Beyond cold somatostatin analogues (SSA), as a first-line antiproliferative drug, peptide receptor radionuclide therapy (PRRT) has emerged as a highly effective treatment in metastatic, well-differentiated GEP-NET of low and intermediate grade G1 and G2 during the last decades [81,82]. In 1992, the first PRRT was carried out in Rotterdam with 111In-DTPA-octreotide (OctreoScan^®^), a tracer well known for functional imaging with short path length and a release of gamma radiation. The first goals, such as a reasonable response and a remarkable suppression of carcinoid symptoms, were achieved [83]. The next generation of beta-emitting agents developed was the 90Y radionuclide conjugated to DOTA peptides (DOTATATE and DOTATOC). Objective response and longer progression-free survival (PFS) could be frequently demonstrated [84], albeit careful monitoring of the kidneys was recommended [85]. Due to a long tissue penetration depth of the 90Y radionuclide (12 mm) and a physical half-life time (t1/2) of 64 h, significant damage to kidney glomeruli and bone marrow was a common side effect. Of the 1109 patients treated with 90Y-DOTATOC, 12.8% developed grade 3 to 4 transient hematologic toxicities, and more than 9% experienced grade 4 to 5 permanent renal toxicity [86]. Therefore, a renal protective regimen of amino acid co-infusion with lysine/arginine was introduced. In recent years, the use of 90Y has been largely replaced by 177Lu (path length 2 mm; t1/2 6.7d) due to considerable advantages. The reduction of minor side effects—such as abdominal pain depending on the location of metastases, and a remarkable decrease of adverse sequelae, such as, nephrotoxicity—to less than 1% was achieved [87]. In addition, 177Lu is also a γ-emitter, which enables visualization on single-photon emission computed tomography (SPECT/CT), staging, and dosimetry [88]. Moreover, Kwekkeboom et al. demonstrated a median PFS of 40 months and an overall survival (OS) of 128 months in 310 patients after somatostatin long acting repeatable (LAR). Patients showed 3% complete response (CR), 28% partial response (PR), and 16% minor response (MR) [87]. Several retrospective studies focusing on feasibility, outcome, and safety of PRRT also showed a PFS of the commonly “hot” somatostatin analog comparable and, in some cases, superior to other treatment modalities (Table 3). Given the fact that these studies were of retrospective design, the results had to be followed up in prospective trials.

**Figure 1:**
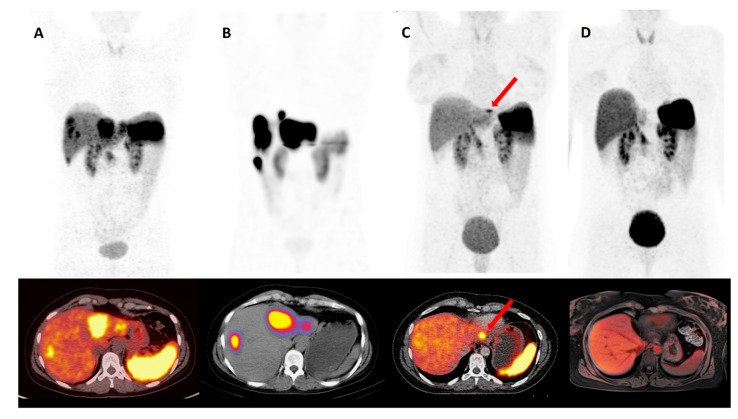
A 38-year-old woman with NET of the rectum G3 (Ki-67 in hotspots up to 25%) with hepatic and locoregional lymph node metastases. Pretherapeutic (**A**) and post-therapeutic (**C**) ^68^Ga-DOTATAOC-PET/CT. After interdisciplinary tumor board decision, 1st cycle PRRT with 7.4 GBq ^177^Lu-DOTATOC (**B**). After three cycles of PRRT, only one remaining hepatic lesion in segment II (**C**, red arrow head) is left. Following a curative approach, the patient underwent a laparoscopic left-lateral liver resection. The patient is currently undergoing semi-annual screening at complete response (CR) ^68^Ga-DOTATAOC-PET/MR (**D**).

The recently published results of NETTER-1, the first prospective randomized study in patients with progressive metastatic midgut NETs, showed a median PFS of 28.4 months after PRRT compared to “high-dose” SSA, with a median PFS of 8.4 months (double dose, 60 mg octreotide LAR every 4 weeks). The OS for the PRRT arm has not yet been reached [93]. By contrast, the current published European Neuroendocrine Tumour Society (ENETS) guidelines recommend the use of PRRT in intestinal (midgut) NETs with distance and/or locoregional metastases as a second- to third-line therapy after progression under SSA, irrespective of the abovementioned NETTER-1 results. According to guidelines in pancreatic NET with advanced locoregional disease, PRRT should even be used as a third line therapy after failure of SSA, everolimus and/or cytotoxic chemotherapy [42]. On this basis, a new phase III study was recently initiated. In the COMPETE study (see clinicaltrials.gov NCT03049189), 177Lu-PRRT is compared with the mTOR inhibitor everolimus in patients with GEP-NET G1 and 2. Despite the success of 177Lu-DOTATATE PRRT, not all patients benefit from PRRT, and patients’ relapse after starting treatment is, on average, seen after 2–3 years [94]. In 2018, a retrospective study of 168 patients with unresectable GEP-NETs treated at the University Hospital Bonn, Germany was published. The patients were divided into two groups: PRRT monotherapy (*n* = 81, group 1) and PRRT plus SSA (*n* = 87, group 2). The results showed a higher median PFS (48 months) in the subgroup receiving the combination therapy, compared to the subgroup receiving only PRRT (27 months) [95]. Accordingly, different experimental approaches and strategies are being explored in order to optimize the effectiveness of PRRT and to minimize potential side effects. First of all, after completing four cycles of PRRT, treatment may even be continued, depending on kidney and bone marrow tolerance, e.g., with reduced radioactivity as part of a salvage therapy (Re-PRRT) [96]. Van der Zwan et al. demonstrated that a cumulative dose of up to 60.5 GBq salvage PRRT with 177Lu-DOTATATE is safe and effective in patients with progressive disease (relapse-PD) after four cycles of 177Lu-DOTATATE PRRT [97]. Furthermore, no increasing incidence of acute myeloid leukemia or myelodysplastic syndrome was observed, and no grade III or IV nephrotoxicity occurred [97]. Further intensification of the PRRT might be even achieved by administration of the tracer directly into the hepatic artery. In particular, patients with hepatic dominant metastases would benefit from this approach due to an increase of uptake of the radiopharmaceutical (the so-called “first-pass” effect) [98]. The PRRT is becoming increasingly important in a neoadjuvant setting [99]. In patients with inoperable P-NET and distant (metastatic) disease, PRRT was associated with a significant reduction in tumor size, and the tumor was rendered operable [100]. In such cases, complete response can be achieved. Figure 1 demonstrates a representative case of PRRT in a neoadjuvant setting.

So far, only SSR agonists are labeled with beta-emitters. SSR antagonists promise a higher binding affinity for somatostatin positive tumor cells, thus leading to an increasing radiation dose within the tumor [101]. Another interesting approach is the use of alpha emitters, such as 213Bismuth (tissue penetration 45μm, t1⁄2 45min) and 225Actinium (tissue penetration 45 μm, t1⁄2 10d), as a targeted alpha particle therapy (TAT) [102]. Treatment with TAT has been gaining popularity over the past few years, especially in the treatment of castration-resistant prostate cancer with 177Lu-prostate-specific membrane antigen (PSMA) [103]. It is hypothesized that the advantage lies in a low tissue penetration depth with high ionizing radiation. A transfer of those promising results to TAT in NETs is the next logical step. However, the evidence for TAT is currently still sparse. Another promising development was reported recently by Zhang et al. regarding the novel SSR agonist DOTA-EB-TATE, also labeled with 177Lu. This agonist showed remarkably higher uptake and retention in NETs, compared to octreotide and octreotate. The authors conclude that in the future, it could possibly be used in PRRT for NETs, with the benefit of lower radiation dose and less frequent administration [104].

## 5. Conclusions

Despite intense research efforts, few patients with neuroendocrine tumors are amendable for curative tumor resection and, for many patients, only palliative treatment options such as SSA or PRRT are available. With this review, we aim at providing a comprehensive overview on the use of SSAs both in an antisecretory and antiproliferative role in patients with NETs. Moreover, we aim at highlighting different experimental strategies (dose escalation, combination therapies, PRRT in early therapy lines) demonstrating how SSA therapy might further evolve in the context of NETs to further improve patient outcome. Finally, we pointed out that many of the available trials and studies suffered from only limited patient numbers and a retrospective and/or monocentric design, underscoring the need for more prospective, randomized phase III trials in the field of NETs. 

## Figures and Tables

**Table 1:** Long-term results from the PROMID trial.

PROMID [68,71]	Octreotide (*n* = 42)		Placebo (*n* = 43)		Total (*n* = 85)
HL	*HL ≤ 10%*	*HL > 10%*	*HL ≤ 10%*	*HL > 10%*	
**Deaths (*n*)**	13/32	9/10	19/32	7/11	48/85
**Median TTP**	14.3 months		6.0 months		
**Median OS**	84.7 months		83.7 months		84.7 months
**Median OS**	Not reached	NA	87.2 months	NA	
**5-YSR**					66.5%
**10-YSR**					45.3%

YSR = year survival rate; HL = hepatic tumor load; OS = overall survival; TTP = time to progression; NA = not available.

**Table 2:** Long-term results from the CLARINET trial (including OLE).

CLARINET (Including OLE) [69,70]	Lanreotide (*n* = 101)	Placebo (*n* = 103)	Total (*n* = 204)
Deaths (*n*)	19/101	17/103	36/204
Median PFS	32.8 months	18.0 months	
AEs			
Total	38	44	82
Treatment related	22	13	35
Severe	11	11	22
Moderate	17	26	43
Mild	9	7	16
Missing	1	0	1

OLE = open label extension; PFS = progression-free survival; AEs = adverse events.

**Table 3:** Retrospective trials on neuroendocrine tumors (NETs) using peptide receptor radionuclide therapy (PRRT) [84,85,87,89,90,91,92]. PFS = progression-free survival; OS = overall survival.

References	Radio Ligand	Number	PFS (month)	OS (month)
Valkema et al. [89]	^90^Y-DOTATOC	58	29	37
Kwekkeboom et al. [87]	^177^Lu-DOTATATE	310	33	46
Bushnell et al. [84]	^90^Y-DOTATOC	90	16	27
Cwikla et al. [85]	^90^Y-DOTATATE	58	17	22
Pfeifer et al. [90]	^90^Y-DOTATOC	53	29	NA
Bodei et al. [91]	^177^Lu-DOTATATE	39	36	NA
Ezziddin et al. [92]	^177^Lu-DOTATATE	74	26	55
